# Effects of a co-bacterial agent on the growth, disease control, and quality of ginseng based on rhizosphere microbial diversity

**DOI:** 10.1186/s12870-024-05347-3

**Published:** 2024-07-08

**Authors:** Xinyue Li, Qun Liu, Yugang Gao, Pu Zang, Tong Zheng

**Affiliations:** 1 College of Chinese Medicinal Materials and Laboratory of Medicinal Plant Cultivation and Breeding of National Administration of Traditional Chinese Medicine, Jilin Agricultural University, Changchun, 130118 China; 2 Institute of Botany, Jiangsu Province and Chinese Academy of Sciences, Jiangsu Key Laboratory for the Research and Utilization of Plant Resources, Nanjing, 2100147 China

**Keywords:** Co-bacterial agent, Ginseng yield, Ginsenoside, Biological control, Pesticide degradation, Microbial community

## Abstract

**Background:**

The ginseng endophyte *Paenibacillus polymyxa* Pp-7250 (Pp-7250) has multifaceted roles such as preventing ginseng diseases, promoting growth, increasing ginsenoside accumulation, and degrading pesticide residues, however, these effects still have room for improvements. Composite fungicides are an effective means to improve the biocontrol effect of fungicides, but the effect of Pp-7250 in combination with its symbiotic bacteria on ginseng needs to be further investigated, and its mechanism of action has not been elucidated. In this study, a series of experiments was conducted to elucidate the effect of *Paenibacillus polymyxa* and *Bacillus cereus* co-bacterial agent on the yield and quality of understory ginseng, and to investigate their mechanism of action.

**Results:**

The results indicated that *P. polymyxa* and *B. cereus* co-bacterial agent (PB) treatment improved ginseng yield, ginsenoside accumulation, disease prevention, and pesticide degradation. The mechanism is that PB treatment increased the abundance of beneficial microorganisms, including *Rhodanobacter*, *Pseudolabrys*, *Gemmatimonas*, *Bacillus*, *Paenibacillus*, *Cortinarius*, *Russula*, *Paecilomyces*, and *Trechispora*, and decreased the abundance of pathogenic microorganisms, including *Ellin6067*, *Acidibacter*, *Fusarium*, *Tetracladium*, *Alternaria*, and *Ilyonectria* in ginseng rhizosphere soil. PB co-bacterial agents enhanced the function of microbial metabolic pathways, biosynthesis of secondary metabolites, biosynthesis of antibiotics, biosynthesis of amino acids, carbon fixation pathways in prokaryotes, DNA replication, and terpenoid backbone biosynthesis, and decreased the function of microbial plant pathogens and animal pathogens.

**Conclusion:**

The combination of *P. polymyxa* and *B. cereus* may be a potential biocontrol agent to promote the resistance of ginseng to disease and improve the yield, quality, and pesticide degradation.

**Supplementary Information:**

The online version contains supplementary material available at 10.1186/s12870-024-05347-3.

## Introduction

Ginseng (*Panax ginseng* C. A. Meyer), a perennial herb genus in the family Araliaceae, is a traditional Chinese herb with significant medicinal and economic value [1]. Its demand is huge and it has a large planting area. Ginseng cultivation methods can be classified as forest-cultivated ginseng, cutting down forest-cultivated ginseng, and farmland-cultivated ginseng [2]. The cultivation of *P. ginseng* in the forest is a kind of wild cultivation mode. With an increase in the growing years, there are problems such as severe disease, long growth cycles, slow ginsenosides accumulation, and high pesticide residues, which seriously affect the yield and quality of ginseng [3, 4], limiting its production and application of ginseng. Beneficial microorganisms play an important role in biological control, plant growth promotion, effective composition accumulation and pesticide residue degradation [5, 6]. The use of bioinoculants for the ecological cultivation of ginseng is of great relevance for high-quality and safe ginseng herbs [7].

Biological control is an environmentally friendly and low-cost ginseng disease control method attracting increasing attention. *Bacillus amyloliquefaciens* FG14 showed good control of ginseng root rot [8]; *Bacillus subtilis* HK-CSM-1 can be used as an effective and ecologically friendly biological control agent for anthracnose in *P. ginseng* [9]; and ginseng endophytic PgBE14 (*Bacillus amyloliquefaciens*), PgBE40 (*B. megaterium*), PgBE45 (*Pseudomonas frederiksbergensis*), and PgBE42 (*Staphylococcus saprophyticus*) are antagonistic to two pathogens *Cylindrocarpon destructans* and/or *Botrytis cinerea* [10]. However, the preventive effect of individual strains is unstable, the spectrum of bacterial inhibition is narrower, and the combined use of bacterial agents to control ginseng diseases is a more reasonable and safer method [11]. The application of corn straw biochar and actinomycetes *Frankia* F1 to prepare composite microbial inoculum results in a better biocontrol effect in ginseng [12]. The combination of bioinoculants is an effective way to control ginseng diseases; however, the combination of more effective microbial agents needs to be screened and the mechanism needs to be clarified.

Microorganisms play an important role in promoting ginseng growth. For example, *Arthrobacter nicotinicola* strain JI39 effectively promotes the growth of ginseng and has the potential to be a good microbial fertiliser for ginseng [13]. Moreover, the endophytic *B. cereus* promotes the growth of ginseng by increasing the content of IAA [14]; The ginseng endophyte *Pseudomonas fluorescens* can also promote the growth of ginseng [15]. The combination of microbial agents has a better growth-promoting effect on ginseng, and the combination of *Bacillus subtilis*, *Bacillus amyloliquefaciens*, and *Paenibacillus mucilaginosus* significantly increases the dry weights of ginseng roots [16]. The combination of biological agents is an effective way to promote the growth of ginseng, but the microbial agents that are more effective in promoting the growth of ginseng needs to be studied, and the associated mechanism needs to be proven.

Microorganisms promote the accumulation of active components in ginseng. *Chaetomium sp.* produces ginsenosides and increases the content of ginsenosides in the adventitious roots of ginseng [17]; inoculation of *Trichoderma citrinoviride* PG87 induced ginsenoside biosynthesis in ginseng plants [18]; and application of *Glomus intraradices* promoted the accumulation of ginsenosides Re, Rg1, Rb1, Rb2, Rc, and Rd in ginseng roots [19]. However, individual strains have limited effects on promoting the accumulation of medicinal components, and a combination of microbial agents has better-promoting effects on the accumulation of ginsenosides. The application of *Bacillus subtilis* and *Trichoderma reesei* bacterial mixture improves the production of ginsenosides in ginseng compared to that with *B. subtilis* or *T. reesei*. [20]. The combination of biological agents is an effective way to promote the accumulation of ginsenosides. However, which agents are more effective in promoting the accumulation of ginsenosides needs to be studied and the underlying mechanism needs to be proven.

In microbial facilitation of pesticides residue degradation, *P. polymyxa* biofertiliser significantly reduces the levels of fluazinam, hexachlorobenzene (BHC), pentachloronitrobenzene (PCNB), chlorpyrifos, and dichlorodiphenyl trichloroethane (DDT) to 66.07%, 46.24%, 21.05%, 72.40%, and 54.21%, respectively, in the roots of ginseng, suggesting that *P. polymyxa* biofertiliser is an effective degrader of this class of pesticides [21]. However, the effects of individual strains on pesticides residue degradation are limited, and a combination of bacterial agents has a stronger effect on the degradation of pesticide residues in ginseng. The hexachlorocyclohexane (HCH) in two different types of HCH-contaminated soils by 53% and 43% respectively after inoculation with compound biofertilizer containing *Rhodococcus erythropolis* ET54b and *Sphingomonas* sp. D4 [22]. The combination of biological agents is an effective way to promote the degradation of ginseng pesticide residues; however, the agents that are more effective in promoting the degradation of ginseng pesticide residues need to be investigated, and the mechanism needs to be elucidated.

The composition and structure of the soil microbial community are closely related to plant growth and health. *Mortierella alpina* has been used as a potential biocontrol agent for *Fusarium oxysporum* to control soil-borne diseases in ginseng by regulating rhizosphere microorganisms [23]. *Bacillus amyloliquefaciens* FS6 reduces the diversity and relative abundance of fungi in the rhizosphere soil of ginseng, which could effectively control seedling diseases and grey mould in ginseng [24]. In this study, we investigated the mechanism by which the combination of biological agents promoted disease resistance, improved yield, quality and pesticide degradation in ginseng through changes in microbial community structure.

Previous studies in our laboratory have shown that *P. polymyxa* Pp-7250 (Pp-7250) can inhibit pathogenic bacteria, promote growth, accumulate active components and degrade pesticide residues in ginseng [25, 26]. *B. cereus* also functions in plant biological control, promoting ginseng growth, and degrading pesticide residues [26–30]. Previous experiments in our laboratory have also shown that *P. polymyxa* and *B. cereus* can undergo symbiotic cultures [31]. However, the effects of the combination of Pp-7250 and *B. cereus* on the biological control, growth promotion, active ingredient accumulation, and pesticides residue degradation in ginseng are not clear, and the underlying mechanism needs to be elucidated. In this study, ginseng seeds and their planted soil were treated with *B. cereus* alone (BC), Pp-7250 alone (PS), and the combined bacteriological agent (PB) to determine the yield, quality, disease prevention of ginseng, and the degradation of organochlorine pesticides in PS, BS, and PB treatments. Differences in the ginseng rhizosphere soil microbial communities among PS, BS, and PB treatments were analysed using high-throughput amplicon sequencing. Through correlation analysis, the mechanism of a combination of microbial agents in promoting growth, biocontrol, ginsenoside content accumulation, and pesticide residue degradation in ginseng was clarified. The results of this study showed that the combination of *P. polymyxa* Pp-7250 and *B. cereus* has better effects than a single microbial agent, providing a theoretical reference for the application of complex microbial agents in ginseng production practice.

## Materials and methods

### Experimental material

*Paenibacillus polymyxa* Pp-7250 (Pp-7250) [25], *Bacillus cereus* strains were isolated [26], identified and preserved in our laboratory. Dormancykreleased ginseng seeds were purchased from Jilin Shenyang Ginseng Co. Ltd. (Jilin, China). Ginsenoside standards Rg1 (110,703–202,129), Re (110,754–202,129), Rf (111,719–201,806), Rb1 (111,779–200,801), Rg2 (110,704–202,129), Rb2 (111,715–201,203), Rb3 (111,686–202,005), Rd (111,818–202,104), purchased from the China Institute of Food and Drug Verification (Beijing, China). Ginsenoside standard Rc (A0243), purchased from Beijing Yihua Tongbiao Science and Technology Co., Ltd.Organochlorine pesticide standards hexachlorobenzene (BHC, GBW (E) 081914), heptachlor (GBW (E) 081455), aldrin (GBW (E) 081457), oxo chlordane (SB-05-380-2017), epoxychlordane (GBW(E)08145), purchased from Beijing Coastal Hongmeng Standard Substance Technology Co., Ltd. Pentachloronitrobenzene (PCNB, 1501), purchased from Dima Science and Technology Co., Ltd. (Beijing, China). Trans-chlordane (SB-05-064-2008), cis-chlordane (SB-05-065-2008), purchased from the Institute of Environmental Protection and Monitoring, Ministry of Agriculture of China. HPLC-grade acetonitrile and methanol were purchased from Fisher (USA).

### Experimental design

The experiment was conducted from April 2023 to October 2023 at the experimental base of Jinda Ginseng Round Co Ltd, Changbai Mountain of Fusong County, Jilin Province, China. The experimental field is an old ginseng field that has been planted for many years, the pesticide residues produced by the long-term application of pesticides. The experiment was designed as four adjacent plots, *B. cereus* alone (BS) and Pp-7250 alone (PS), Pp-7250 in combination with *B. cereus* (PB) and sterile water control (CK) were applied, respectively, with three replicates in each group and each replicate area of 3 m^2^ (2 m length × 1.5 m width). Among them, the ratio of Pp-7250 and *B. cereus* in the PB treatment group was 1:1. On 25 April 2023, ginseng seeds were surface sterilised with 1% sodium hypochlorite for 5 min, rinsed with sterile water 3–5 times. Seeds in each treatment group were sprayed with 30 ml (10^6^cfu/ml) of each bacterial solution and sterile water correspondingly, and 35 seeds per row at 15 g of seed per 3 m^2^. Ginseng seedlings in each group were sprayed with 150 ml (2 × 10^5^cfu/ml) of each bacterial solution and sterile water on 20 June and 20 July 2023, respectively.

### Ginseng and soil sample collection

On 15 October 2023, three sampling points (3 rows) were taken from each treatment group, each sampling point used a standard sample area (approximately 0.15 m^2^ per sample) of ginseng plants, the rhizosphere soil was collected according to Riley and Barber’s standards [32], and passed through a 2 mm sieve, the treated samples were stored at -80 °C for microbiome analysis.

### Determination of ginseng disease index

Surviving ginseng plants were counted and seedling-preservation rate was calculated. Observe and record the number of plants with ginseng root and leaf disease, and calculate the plant incidence.The formula is as follows: Disease incidence (%) = (number of diseased plants / total number of plants) × 100. Calculation of relative biological defence effectiveness based on incidence rates, the formula is as follows: Relative efficacy (%) = ((control incidence - treatment incidence) / control incidence) × 100.

### Determination of ginseng growth potential

The collected ginseng plants were separated into roots, stems and leaves. Ginseng plant height (cm), petiole length (cm), stem diameter (mm), leaf length (cm), leaf width (cm), root length (cm), root diameter (mm) were measured by Vernier caliper. Ginseng plant fresh weight (g), root fresh weight (g), stem fresh leaves weight (g) was measured with an analytical balance to compare the differences in growth potential among treatments.

### Determination of ginseng pesticide residue

Sample pre-treatment and GC determination are based on the methods used in our laboratory [21]. Gas chromatography (GC-14C) equipped with Nickel-63 electron capture detector (ECD) supplied by Shimadzu, Japan. Capillary column Agilent DB-1701 (30 m × 0.25 mm, 0.25 μm) was used for detecting. Carrier gas (N2, 99.999% purity) flow was maintained 1 mL min^− 1^ with split ratio 10:1. The working conditions of GC were as follows: injector temperature 260 °C, and detector temperature 300 °C. The column initial temperature 60 °C held for 3 min, increased to 170 °C at a rate of 10 °C min^− 1^ and held for 20 min, increased to 260 °C at a rate of 10 °C min^− 1^ and held for 10 min, increased to 240 °C at a rate of 1 °C min^− 1^ and held for 15 min, increased to 280 °C at a rate of 15 °C min^− 1^ and held for 5 min. Injection volume 1 µL.

### Determination of ginsenoside content

Sample pre-treatment and HPLC determination are based on the methods used in our laboratory [33]. 9 ginsenosides were determined by high performance liquid chromatography (HPLC, LC-2010A) with C18 column (250 mm × 4.6 mm, 5 μm) supplied by Shimadzu, Japan. The column temperature was maintained at 35 °C. The binary mobile phase consisted of water (W) and acetonitrile (AN). Gradient elution procedure: 0–24 min, 18% AN, 82% W; 24–26 min, 22% AN, 78% W; 26–30 min, 26% AN, 74% W; 30–50 min, 32% AN, 68% W; 50–55 min, 33.5% AN, 66.5% W; and 55–65 min, 38% AN, 62% W. The flow rate was maintained at 1.0 mL/min and the absorbance was measured at a wavelength of 203 nm for ginsenoside detection. Injection volume: 10 µL.

### Analysis of microbiota diversity in ginseng rhizosphere soil

#### Genomic DNA preparation, PCR amplification and Illumina MiSeq sequencing

Total DNA was extracted from 0.5 g of each rhizosphere soil sample using the SPINeasy soil DNA Kit (MP Biomedicals, LLC, USA) according to the manufacturer’s protocol. 1% agarose gels and Nano Drop 2000 (Thermo Fisher Scientific, MA, USA) were performed for the DNA concentration and purity control. Broad-spectrum primer sets 338 F (5 ' -ACT CCT ACG GGA GGC AGC A-3 ‘) -806R (5 ' -GGA CTA CHV GGG TWT CTA AT-3 ‘), and ITS1F (5’-CTTGGTCATTTAGAGGAAGTAA-3’) -ITS2 (5’- GCTGCGTTCTTCATCGATGC-3’) amplification of the 16S rRNA V3 + V4 region and the RNA manipulator ITS region of each sample in combination with adapter sequences and barcode sequences were used to evaluate soil bacterial and fungi community [34]. The capacity of each PCR reaction system was 20 µL containing 4 µL 5 × FastPfu Buffer, 2 µL 2.5 mM dNTPs, 0.8 µL 5 µM primers, 0.4 µL FastPfu Polymerase and 10 ng DNA template. PCR conditions included an initial denaturation at 95 °C for 5 min, 25 cycles of 95 °C for 30 s, 55 °C for 30 s, and 72 °C for 40 s; and a final extension step at 72 °C for 7 min. PCR products were quantified using an enzyme marker and combined at 1:1 ratio. DNA was purified using an OMEGA DNA Purification Kit (Omega, USA). The library was tested using Qsep-400, Illumina novaseq6000 was used for sequencing after quality inspection of the library to obtain 250 bp paired-end raw reads. DNA library construction and sequencing were conducted by the Biomarker Company. (Biomarker Technologies Corporation, Beijing, China).

#### Bioinformatics analysis

Raw data were primarily filtered by Trimmomatic (version. 0.33) [35]. Then, primer sequence recognition and removal were performed for high-quality reads by using Cutadapt software (version. 1.9.1) [36], the clean reads were spliced using Usearch software (v.10) [37], high-quality sequences were obtained for subsequent analyses, and the sequences were finally obtained with high quality for subsequent analysis. The detailed quality control information appears in Table S1, 2. Sequences were analysed using the QIIME 2 package (https://qiime2.org/), the sequences were clustered at 97% level of similarity, and 0.005% of the number of sequences were used as the threshold to filter OTUs. Representative sequences of each OTU were screened for further annotation. For each representative sequence, the Silva Database (Release. 132, http://www.arb-silva.de) and Unite (Release. 8.0, https://unite.ut.ee/) were used based on Mothur algorithm to annotate taxonomic information. To study the phylogenetic relationship of different OTUs, and the difference of the dominant species in different samples (groups). Multiple sequence alignment was performed with MUSCLE (v.3.8.31, http://www.drive5.com/muscle/). The OTUs abundance information was based on the sample with the least sequences.

Rarefaction curves and Shannon curves were plotted using R software (v.3.6.0). Venn diagrams were plotted using the R package VennDiagram (https://cran.r-project.org/web/packages/VennDiagram/index.html). Alpha diversity indices were calculated using QIIME2. (https://qiime2.org/), the one-way ANOVA test was sed to evaluate the difference in alpha diversity across sample compartments, GraphPad Prism (v.8.0) plotted box plots. PCoA and NMDS were used to show the Differences in microbial community structure among the four treatments [38]. Hierarchical clustering heatmap was visualized using the MeV 4.9.0 software. Mantel tests and Permutational ANOVAs (Permanovas) were performed to assess the correlation between rhizosphere microbial communities and ginseng growth index, disease resistance level by using R package ‘vegan’ (v. 2.5-7), respectively [39]. Redundancy analysis (RDA) was performed via Canoco for Windows 5 (Microcomputer Power, NY, USA) [40]. Microbial functional profiles were predicted using the PICRUSt2 (https://github.com/picrust/picrust2) bacterial database and the FUNGuild (https://www.funguild.org/) fungi database.

### Statistical analysis

Statistical analyses and graphical plotting of data on ginseng growth indices, disease prevention levels, ginsenoside levels, degradation of pesticides, soil microbial α-diversity, generic level differences, and functional differences were performed using SPSS (v. 26.0), GraphPad Prism (v. 8.0), and Origin (v. 2021). All values were stated as means ± standard deviation (SD), and z-score was used to normalise the data. Statistical comparison was carried out with Students *t*-test or one-way analysis of variance (ANOVA), the statistical significance was set at *p < 0.05, **p < 0.01.

## Results

### Reduction of ginseng disease incidence by application of Pp-7250 co-bacterial agent

**Fig. 1:**
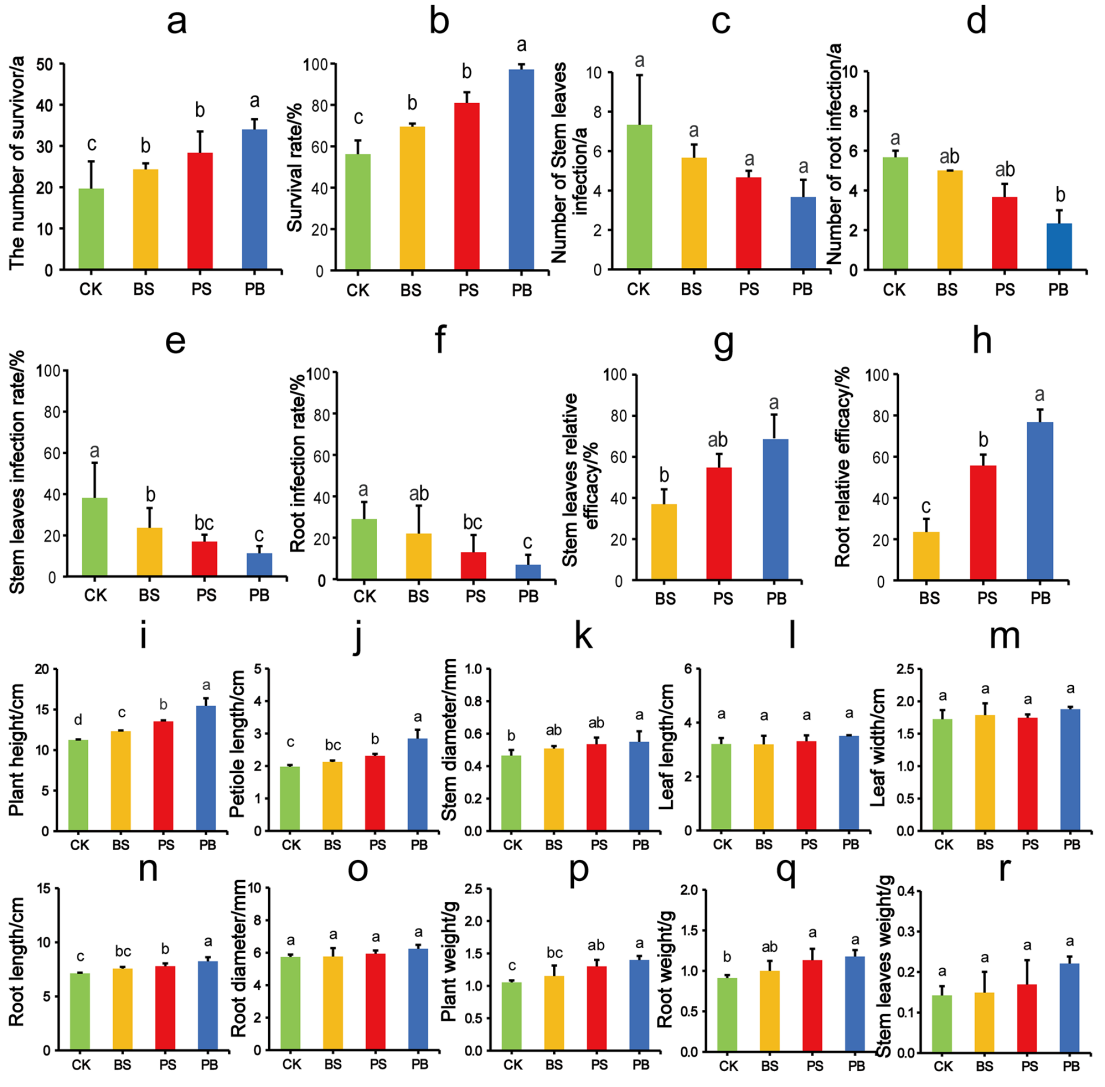
Effect of Pp-7250 co-bacterial agent on ginseng disease incidence (**a-h**) and Ginseng agronomic traits (**i-r**). A Different letters indicate significant differences between treatments, *p* < 0.05)(*n* = 3). CK: Blank control group, BC: *B. cereus* treatment group, PS: Pp-7250 treatment group, PB: Pp-7250 co-bacterial agent treatment group

According to the experimental results, the number of survivors and survival rates in the group PB were significantly higher than those in the group PS, PS group was significantly higher than that in BS group, and BS group was significantly higher than that in group CK (*p* < 0.05). The survival rate of the control group without bioinoculants was only 56.19%. Significantly increased survival rates after treatment with *B. cereus* and Pp-7250 (BS: 13.33%, PS: 24.76%, *p* < 0.05), and the survival rate was as high as 97.14% after Pp-7250 co-bacterial agent treatment. Simultaneous application of microbial agents (PS and PB) significantly reduced the rate of ginseng root, stemsand leaves infections (*p* < 0.05), thereby increasing their relative efficacy. Relative efficacy against BS, PS, and PB roots was 23.45%, 55.66%, 76.76% (*p* < 0.05), and relative efficacy against stem and leaves was 36.95%, 54.82%, 68.39% (Fig. 1a-h, Table S3). Bioinoculants application significantly affected the growth and health of ginseng.

### Growth promotion of ginseng by application of Pp-7250 co-bacterial agent

Significant effects of Pp-7250 co-bacterial agent on agronomic traits of ginseng (Fig. 1i-r, Table S4). Plant height was significantly higher in group PB than in group PS, those of group PS was significantly more than in group BS, which was significantly more than in group CK (*p* < 0.05). Root length and petiole length were significantly higher in group PB than in group PS, and in group PS were significantly higher than those in groups BS and CK (*p* < 0.05). Plant weight was significantly higher in group PB than in groups BS and CK, and in group PS was significantly higher than those in group CK (*p* < 0.05). Root weight was significantly higher in groups PS and PB than in groups BS and CK. Stem diameter was significantly higher in group PB than in groups CK (*p* < 0.05). Root diameter, leaf length, leaf width and stem and leaf weight were not significantly different between the groups (*p* < 0.05). Plant height, root length and root weight increased by 37.07%, 15.71% and 29.67%, respectively, in group PB compared with the group CK. Agronomic traits are important indicators for evaluating plant growth characteristics, suggesting that Pp-7250 co-bacterial agent promoted the growth of 1-year-old ginseng.

### Promotion of ginsenoside accumulation by application of Pp-7250 co-bacterial agent

**Fig. 2:**
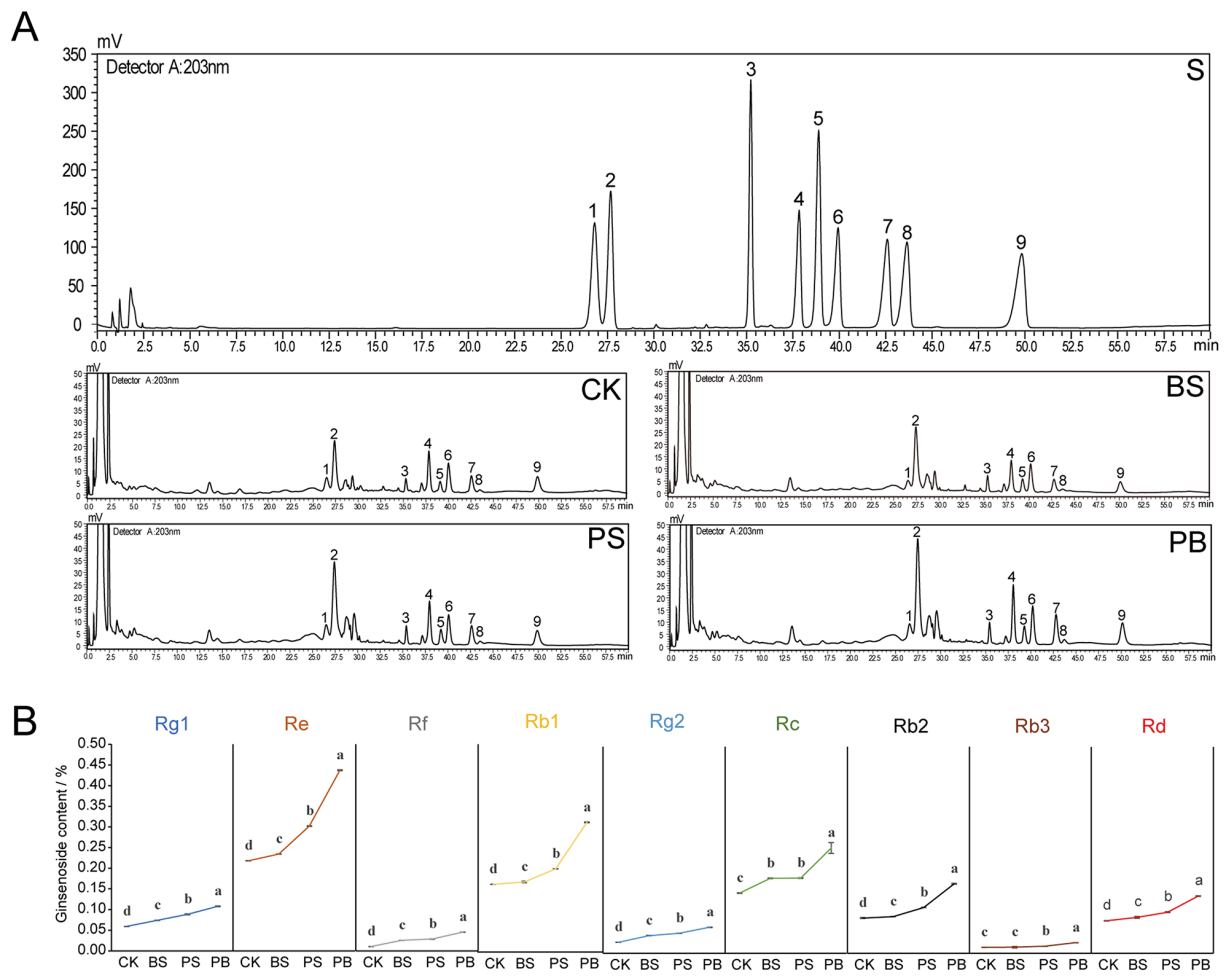
Effect of Pp-7250 co-bacterial agent on accumulative of 9 ginsenosides (*n* = 3). (**A**) High-performance liquid chromatogram of ginsenosides detected from ginseng. (**B**) Content of ginsenoside (%). Different letters indicate significant differences between treatments, *p* < 0.05. S: mix control, CK: blank control group, BS: *B. cereus* treatment group, PS: Pp-7250 treatment group, PB: Pp-7250 co-bacterial agent treatment group. 1–9: Rg1, Re, Rf, Rb1, Rg2, Rc, Rb2, Rb3, Rd

By determining the contents of nine major ginsenosides in ginseng under the four treatments (Fig. 2, Table S5), the contents of Rg1, Re, Rf, Rb1, Rg2, Rb2 and Rd were PB > PS > BS > CK (*p* < 0.05), which showed a similar pattern. The content of Rc was significantly higher in group PB than in the other three groups, the content of Rb3 was significantly higher in groups PB and PS than in the other two groups, and the contents of the nine ginsenosides in group PB were 1.25, 2.00, 4.35, 1.91, 2.37, 1.75, 1.97, 1.79, and 1.76 times higher than that of the control respectively. We speculate that application of biofertilizers increase the ginsenoside content, and the application of Pp-7250 co-bacterial agent was more helpful for the accumulation of ginsenosides. Based on the results of the study, co-bacterial agent may effectively promote the accumulation of ginsenosides, however, the mechanism of its promotion of ginsenoside accumulation needs to be further explored.

### Promoting the degradation of ginseng pesticide residue by application of Pp-7250 co-bacterial agent

**Fig. 3:**
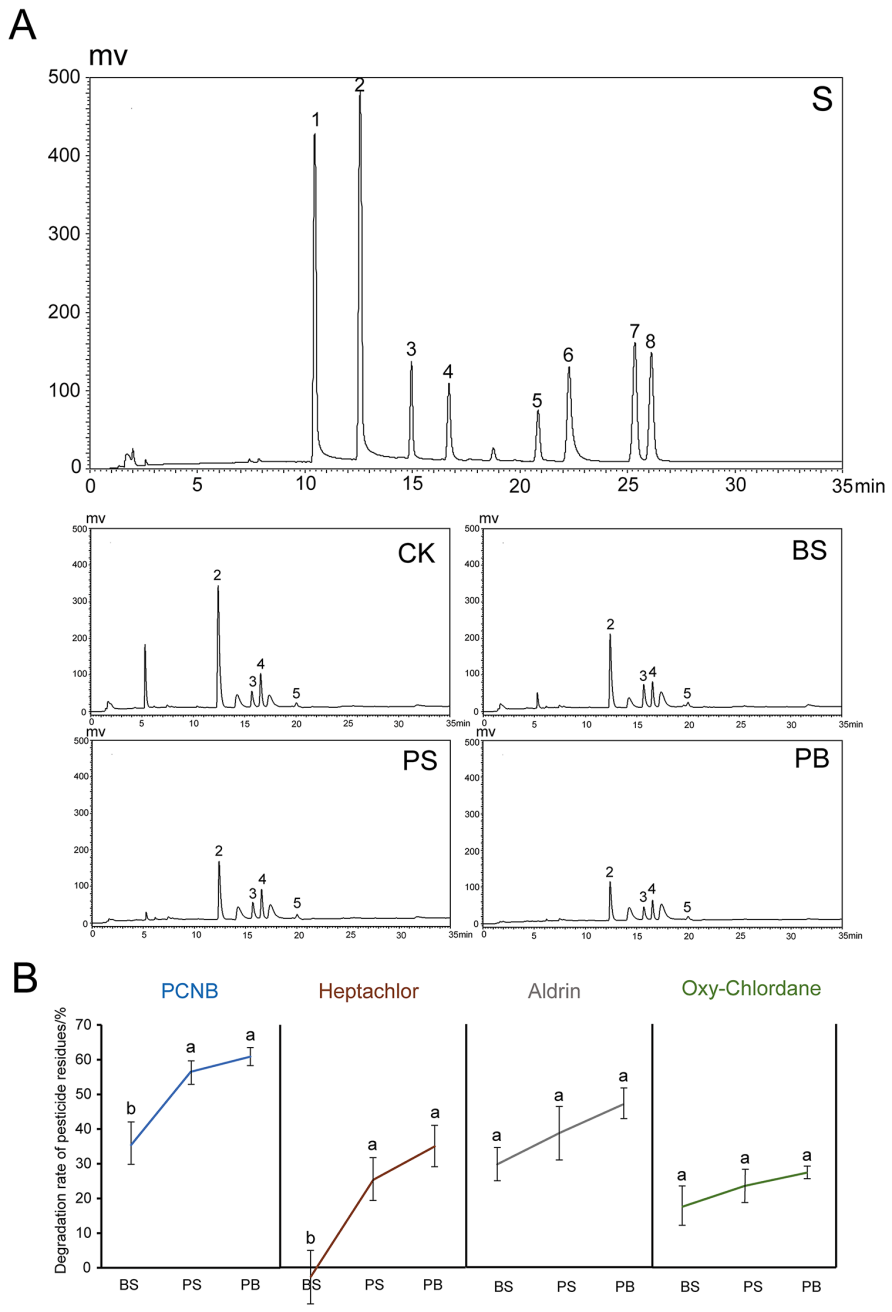
Effect of Pp-7250 co-bacterial agent on the degradation of pesticides residue in ginseng (*n* = 3). (**A**) Gas chromatography of pesticides detected from ginseng. (**B**) Degradation rate of pesticide residues (%). Different letters indicate significant differences between treatments, *p* < 0.05. S: mix control, CK: blank control group, BS: *B. cereus* treatment group, PS: Pp-7250 treatment group, PB: Pp-7250 co-bacterial agent treatment group. 1–8: BHC, PCNB, heptachlor, aldrin, oxo-chlordane, epoxychlordane. trans-chlordane, cis-chlordane

In order to test the ability of microorganisms to degrade organochlorine pesticides, especially the effect of the combination of Pp-7250 co-bacterial agent on the efficiency of pesticides residue degradation in ginseng plants. We determined the levels of eight the Chinese pharmacopoeia organochlorine ginseng pesticide residue under different treatments (Fig. 3, Table S6, 7), of which four organochlorine pesticides, namely, BHC, epoxychlordane, trans-chlordane and cis-chlordane, were not detected, while PCNB, heptachlor, aldrin, and oxo-chlordane were detected. The degradation efficiency of PCNB was the most significant, which was significantly higher in groups PB and PS than in group CK by 24.15% and 22.45%, respectively (*p* < 0.05), and the degradation rate of heptachlor in group PB was also higher at 34.15% (*p* < 0.05). Aldrin and oxo-chlordane oxide were also degraded to some extent in the different treatment groups, the degradation rates were not significant. It indicates that Pp-7250 co-bacterial agent has high degradation efficiency for PCNB and heptachlor, however, the degradation mechanism needs to be analysed in subsequent experiments.

### Alteration of ginseng rhizosphere soil microbial by application of Pp-7250 co-bacterial agent

#### Processing of Illumina MiSeq sequencing data

**Fig. 4:**
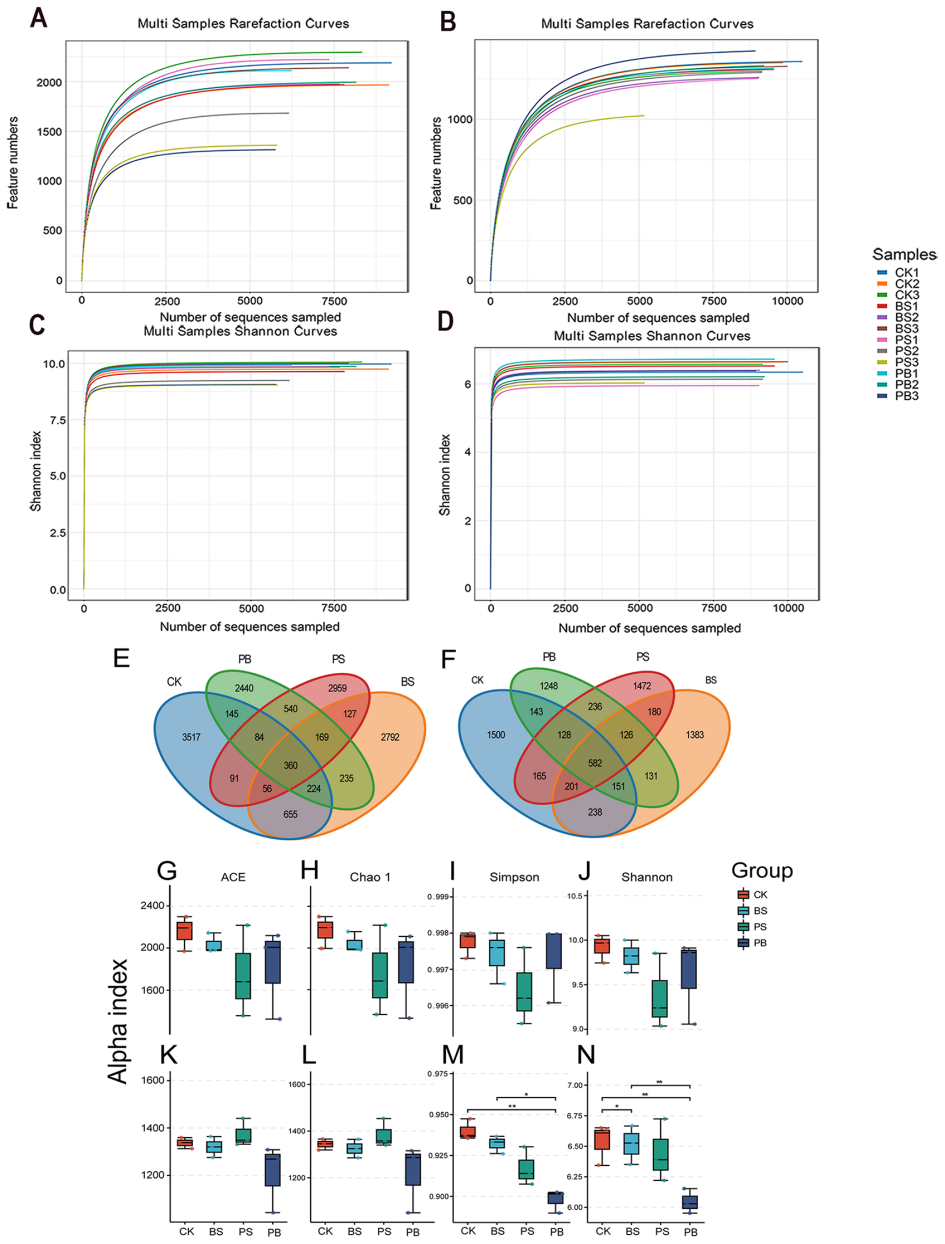
Rarefaction curves, Shannon curves, Venn diagram and alpha diversity index boxplots of bacterial and fungi. (**A**) Rarefaction curves for bacteria (**B**) Rarefaction curves for fungi, (**C**) Shannon curves for bacteria (**D**) Shannon curves for fungi, (**E**) Venn diagrams for bacteria (**F**) Venn diagrams for fungi, (**G-J**) ACE index, Chao 1 index, Simpson index, Shannon index for bacteria, (**K-N**) ACE index, Chao 1 index, Simpson index, Shannon index for fungi

After quality control filtering, the 12 soil samples yielded a total of 1,215,656 valid reads of the V3–V4 region of 16 S rRNA genes, 1,138,162 valid reads in the ITS fungi region, and at least 60,000 (69,402) high-quality bacterial and 50,000 (55,289) high-quality fungi sequences in each replicate. These sequences were grouped into 14,394 bacterial and 7884 fungi OTUs. with an average length of 415 and 242 bp, respectively, using an identity threshold of 97% (Table S1, 2). We used rarefaction curves to evaluate saturation. The results showed that all rarefaction curves increase with the number of sequences, with OTU richness of bacterial and fungi communities almost approached saturation, indicating that the sequencing capacity and the number of sequencing reads were broad enough to capture the complete diversity of these communities (Fig. 4A, B). The Shannon curve indicated that is experimental sample had covered the majority of microbial species information and the amount of sequencing data was sufficiently large (Fig. 4C, D).

#### Microbial diversity of ginseng rhizosphere soil treated with Pp-7250 co-bacterial agent

OTUs of bacteria groups BS, PS, PB, and CK were 4618, 4386, 4197, and 5132, 360 OTUs were common to all samples, OTUs unique to groups PS and PB is 2440, 2959 (Fig. 4E). OTUs of fungi groups BS, PS, PB and CK were 2992, 3070, 2745, 3108, 582OTUs were common to all samples, 1472, 1248 unique to the groups PS and PB (Fig. 4F). To further investigate the composition communities, all bacterial and fungi sequences were classified from phylum to species level (Table S8, 9).

The number of valid sequences and OTUs of the samples were significantly different between the BS, PS, and PB treatment groups and the CK control group (*p* < 0.05) (Fig. 4G-N). Based on 16 S rRNA sequences analysis, at 97% similarity level, it can be seen that the diversity and richness of bacteria in ginseng rhizosphere soil were reduced sequentially in the groups CK, BS, PS, and PB, but the differences were not significant (*p* > 0.05) (Fig. 4G-J). Fungi ITS rDNA sequence analysis, Shannon index in groups PB and BS were lower than those in group CK (*p* < 0.05), and in group PB was significantly lower than in group BS (*P* < 0.05). Simpson index in group PB were both significantly lower than in groups CK and BS (*p* < 0.05) (Fig. 4K-N). It suggests that the Pp-7250 co-bacterial agent inhibited the growth of fungi in the soil, thus reducing the species diversity of the ginseng rhizosphere soil microbial communities, but further analysis is required to determine which fungi were reduced.

#### The difference in ginseng rhizosphere soil microbial communities treated with Pp-7250 co-bacterial agent

**Fig. 5:**
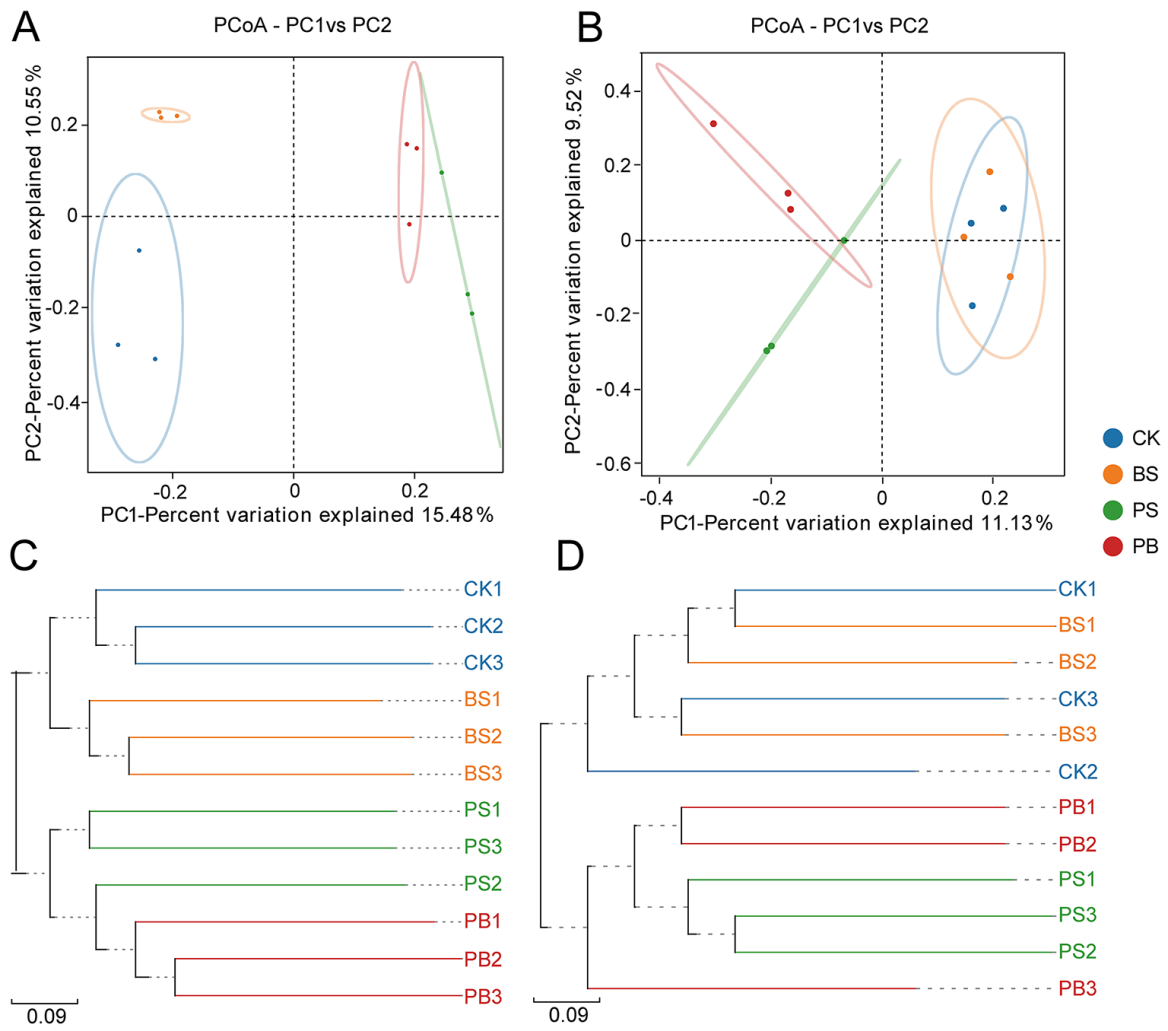
Analysis of the difference in ginseng rhizosphere soil microbial communities treated with Pp-7250 co-bacterial agent. Bacterial community principal coordinate analysis (PCoA) and hierarchical clustering were calculated using the OTU Binary-jaccard distance matrix, fungi community PCoA and hierarchical clustering were calculated using the OTU Bray-Curtis distance matrix (97% OTU cutoff). (**A**) PCoA of bacterial communities, (**B**) PCoA of fungi communities, (**C**) Hierarchical clustering of bacterial communities, (**D**) Hierarchical clustering of fungi communities

PCoA analyses of the bacterial communities showed significant differences between groups, and the differences between groups were greater than that within groups (*r* > 0, *p* < 0.05). In the results of PCoA analysis based on bacterial OTUs, it can be seen that the bacterial communities in both PS and PB treatment groups were significantly separated from the CK and BS groups, as well as between the PS and PB groups, and significantly between the BS and CK groups (Fig. 5A). Combined with UPGMA cluster analysis, microbial agents BS, PS, and PB all had significant effects on soil microbial communities, and microbial agents PS and PB had greater effects (Fig. 5C). The differences in fungi Community Structure Similar to the bacterial community results, but there was a partial overlap between the CK and BS groups (Fig. 5B, D). These results suggested that the microbial communities have significantly changed after inoculation with different microbial agents.

#### Microbial community composition and dominant communities in ginseng rhizosphere soil microbial communities treated with Pp-7250 co-bacterial agent

**Fig. 6:**
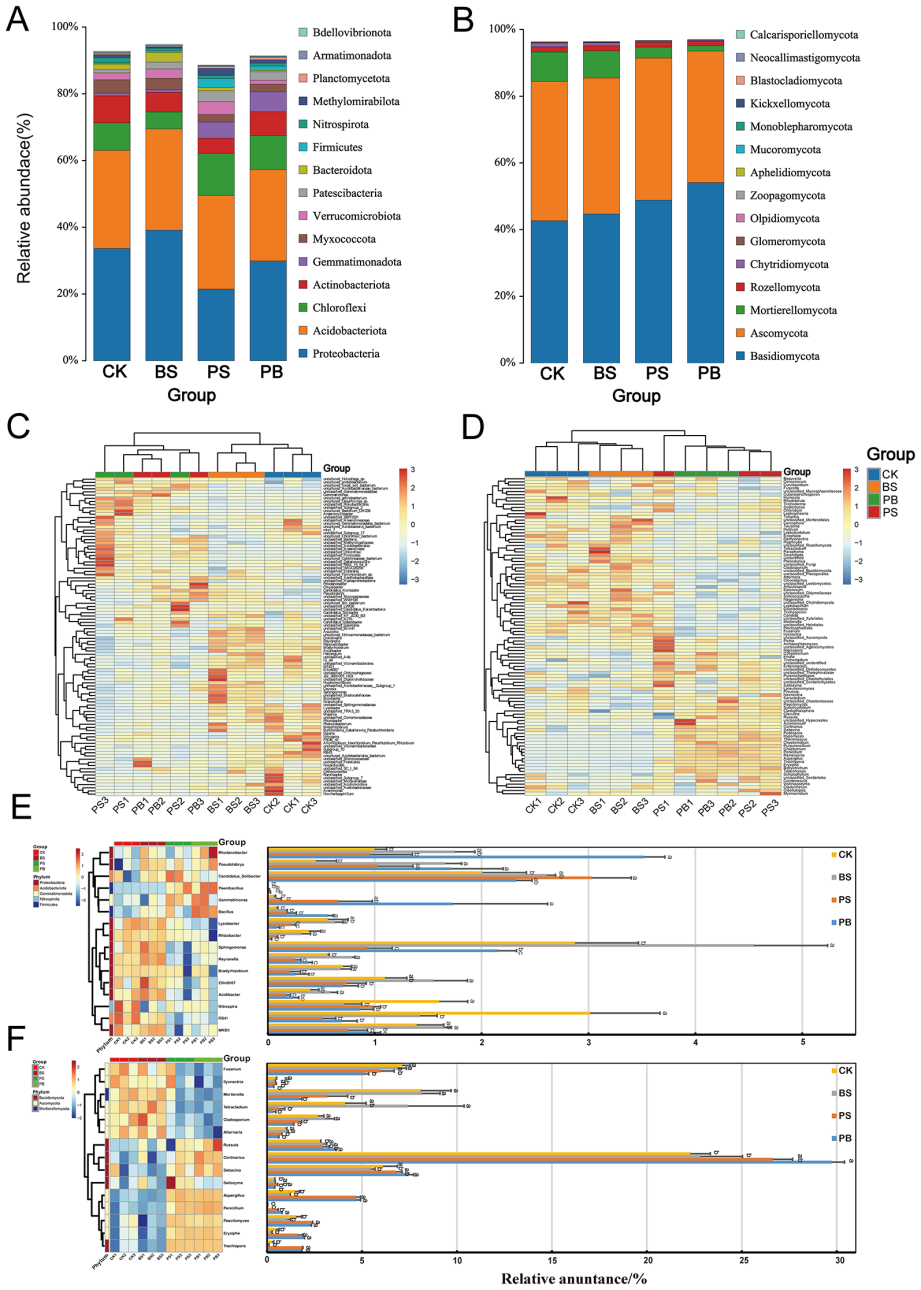
Analysis of soil microbial community composition and dominant communities among different treatments. (**Α. B**) Cumulative histograms of bacterial and fungi community composition (relative abundance (%) of ASVs at the phylum level), (**C. D**) The cluster heatmap of the top 80 genus between the four treatments, (**E. F**) The cluster heatmap of relative abundance of genus with significant differences (ANOVA) and significant difference analysis bar charts between groups of bacterial and fungi (*p* < 0.05)(*n* = 3)

Microbial taxa were identified on the basis of Amplicon Sequence Variant (ASV). Relative abundance of the top 15 bacterial community at the phylum level in the ginseng rhizosphere soil (Fig. 6A, Table S10). Among them, *Proteobacteria* and *Acidobacteria* were the dominant bacterial taxa in the ginseng rhizosphere soil, with relative abundances of 49.31–69.49%. The relative abundance of *Gemmatimonadota* (5.65%) and *Firmicutes* (1.34%) in the group PB was significantly higher than that of the group CK (*P* < 0.05), and the relative abundance of *Chloroflexi* (10.33%, 12.99%) in the groups PB and PS was higher than that of the other treatment groups. *Proteobacteria* (29.94%, 21.29%) had lower abundance in groups PB and PS than in groups BS and CK (*p* < 0.05).

Relative abundance of the top 15 fungi community at the phylum level in the ginseng rhizosphere soil (Fig. 6B, Table S11), *Basidiomycota* and *Ascomycota* were the dominant fungi taxa in the ginseng rhizosphere soil, with relative abundances of 84.40 ~ 93.49%. The relative abundance of *Basidiomycota* (54.10%) in the group PB was significantly higher than that of the group CK (*p* < 0.05), and *Ascomycota *(39.39%) was the lowest in the group PB (*p* > 0.05). The relative abundance of *Mortierellomycota* and *Chytridiomycota* in the groups PB and PS was significantly lower than that of the group CK (*p* < 0.05).

Based on abundance clustering heatmap of the top 80 bacterial community genus in ginseng rhizosphere soil and Table of the top 20 genus (Fig. 6C, E, Table S12). Among the bacterial genus with significant differences between groups were *Sphingomonas*, *Rhodanobacter*, *Candidatus_Solibacter*, *RB41*, *Gemmatimonas*, *Pseudolabrys*, *MND1*, *Nitrospira*, *Ellin6067*, *Reyranella*, *Bradyrhizobium*, *Lysobacter, Acidibacter*, *Bacillus*, *Rhizobacter*, etc., and *Paenibacillus* outside the top 20. The results of ANOVA analysis showed that, *Rhodanobacter*, *Gemmatimonas*, *Pseudolabrys*, *Bacillus*, and *Paenibacillus* had the highest abundance in the group PB, which increased by 252.36%, 3147.28%, 279.47%, 602.49%, and 1537.07%, respectively, compared with CK (*p* < 0.05). Whereas *Ellin6067* had the lowest abundance in the group PB, decreasing by 40.50%, compared to the group CK (*p* < 0.05). Furthermore, *Candidatus_Solibacte* (1.03%) in the group PS obtained significant enrichment (*p* < 0.05). Differences between the groups BS and CK were small.

Based on abundance clustering heatmap of the top 80 fungi community genus in ginseng rhizosphere soil and Table of the top 20 genus (Fig. 6D, F, Table S13). The fungi genus with significant differences among different treatment groups were *Cortinarius*, *Mortierella*, *Sebacina*, *Tetracladium*, *Fusarium*, *Aspergillus*, *Russula*, *Cladosporium*, *Paecilomyces*, *Erysiphe*, *Trechispora*, *Alternaria*, *Saitozyma*, *Ilyonectria*, *Penicillium*, etc. The abundance of *Cortinarius*, *Russula*, *Paecilomyces*, *Trechispora* in the group PB increased by 33.42%, 25.34%, 46.47%, 608.37% compared to the group CK (*p* < 0.05), whereas *Fusarium*, *Tetracladium*, *Alternaria*, and *Ilyonectria* had the lowest abundance in the group PB, which decreased by 38.13%, 112.15%, 70.78%, and 45.23% (*p* < 0.05), respectively, compared to the group CK. *Saitozyma* (0.51%) in the group PS obtained significant enrichment (*p* < 0.05). Differences between the groups BS and CK were small. These results indicated that Pp-7250 co-bacterial agent significantly affected the microbial community in the rhizosphere soil of ginseng, promoting the reduction of some beneficial microorganisms and the increase of some harmful microorganisms.

### Correlation analysis of rhizosphere soil microbial with disease index, agronomic traits, efficacy components and pesticides residue in ginseng treated with Pp-7250 co-bacterial agent

**Fig. 7:**
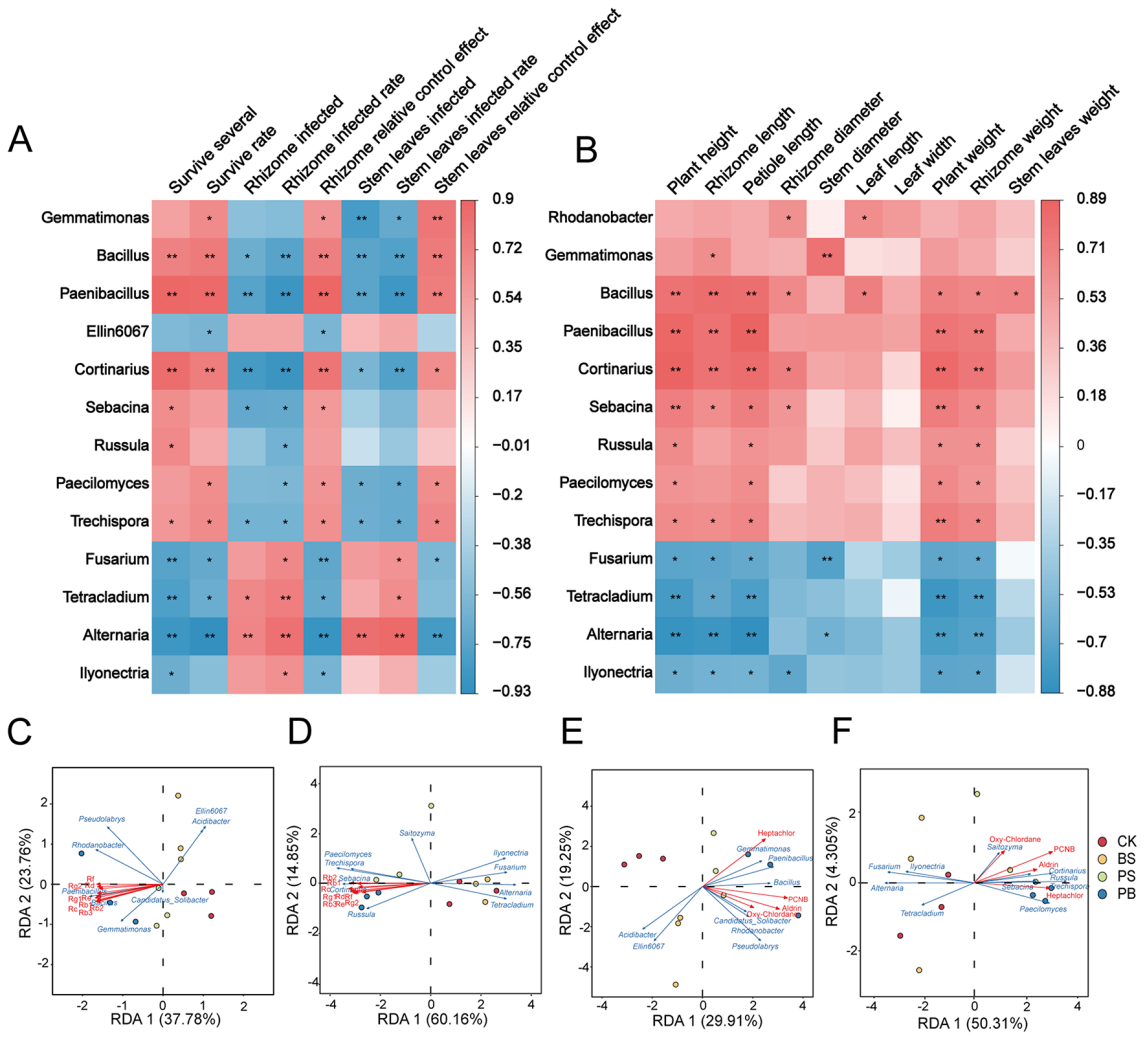
Correlation analysis of ginseng rhizosphere soil microorganisms with disease index (**A**), agronomic traits (**B**), pharmacodynamic components (**C. D**), and pesticide residue (**E. F**). RDA analysis of pharmacodynamic components with key bacterial (**C**) and fungi (**D**), RDA analysis of pesticide residue with key bacterial (**E**) and fungi (**F**) (*p < 0.05; ** *p* < 0.01). CK: blank control group, BC: *B. cereus* treatment group, PS: Pp-7250 treatment group, PB: Pp-7250 co-bacterial agent treatment group (*n* = 3)

We conducted the Pearson correlation analysis to predict the relationship between microorganisms and disease indices, agronomic traits, efficacy components, and pesticide residue. The Pearson correlation coefficients showed that ginseng survival rates and disease prevention were significant positively correlated with the beneficial microorganism *Gemmatimonas*, *Bacillus*, *Paenibacillus*, *Cortinarius*, *Paecilomyces*, and *Trechispora* (*p* < 0.05), and were significant negatively correlated with the fungal genera *Fusarium*, *Mortierella*, *Tetracladium*, *Alternaria*, and *Ilyonectria* (*P* < 0.05) (Fig. 7A). Ginseng plant length and root weight were significant positively correlated with the beneficial microorganism *Bacillus*, *Paenibacillus*, *Cortinarius*, *Sebacina*, *Russula*, *Paecilomyces*, and *Trechispora* (*p* < 0.05), and with the fungal genera *Fusarium*, *Tetracladium*, *Alternaria*, and *Ilyonectria* were significant negatively correlated (*p* < 0.05) (Fig. 7B). It was shown that ginseng diseases and growth are related to the species of rhizosphere soil microbial, and that Pp-7250 co-bacterial agent to increase the beneficial bacteria and reduce the harmful bacteria is a good strategy to improve ginseng production performance.

According to RDA analysis, nine ginsenoside monomers in ginseng in each treatment group showed significant positive correlation with group PB. The nine ginsenoside monomers showed significant positive correlation with the beneficial bacteria *Bacillus*, *Paenibacillus*, and *Cortinarius* in group PB (Fig. 7C, D). It indicated that Pp-7250 co-bacterial agent promoted ginsenoside accumulation by enriching ginseng rhizosphere soil beneficial microorganisms *Bacillus*, *Paenibacillus*, and *Cortinarius*. The degradation rates of pesticides PCNB, heptachlor and aldrin in ginseng showed significant positive correlation with group PB, PCNB and heptachlor showed significant positive correlation with the beneficial bacteria *Paenibacillus* and *Cortinarius* (*p* < 0.01), and aldrin showed significant positive correlation with *Paenibacillus* (*p* < 0.01) (Fig. 7E, F). It indicated that Pp-7250 co-bacterial agent promoted the degradation of ginseng pesticide residue by enriching by enriching ginseng rhizosphere soil beneficial microorganisms *Bacillus*, *Paenibacillus*, and *Cortinarius*.

### Microbial community function in ginseng rhizosphere soil treated with Pp-7250 co-bacterial agent

#### Bacterial community function in ginseng rhizosphere soil treated with Pp-7250 co-bacterial agent

**Fig. 8:**
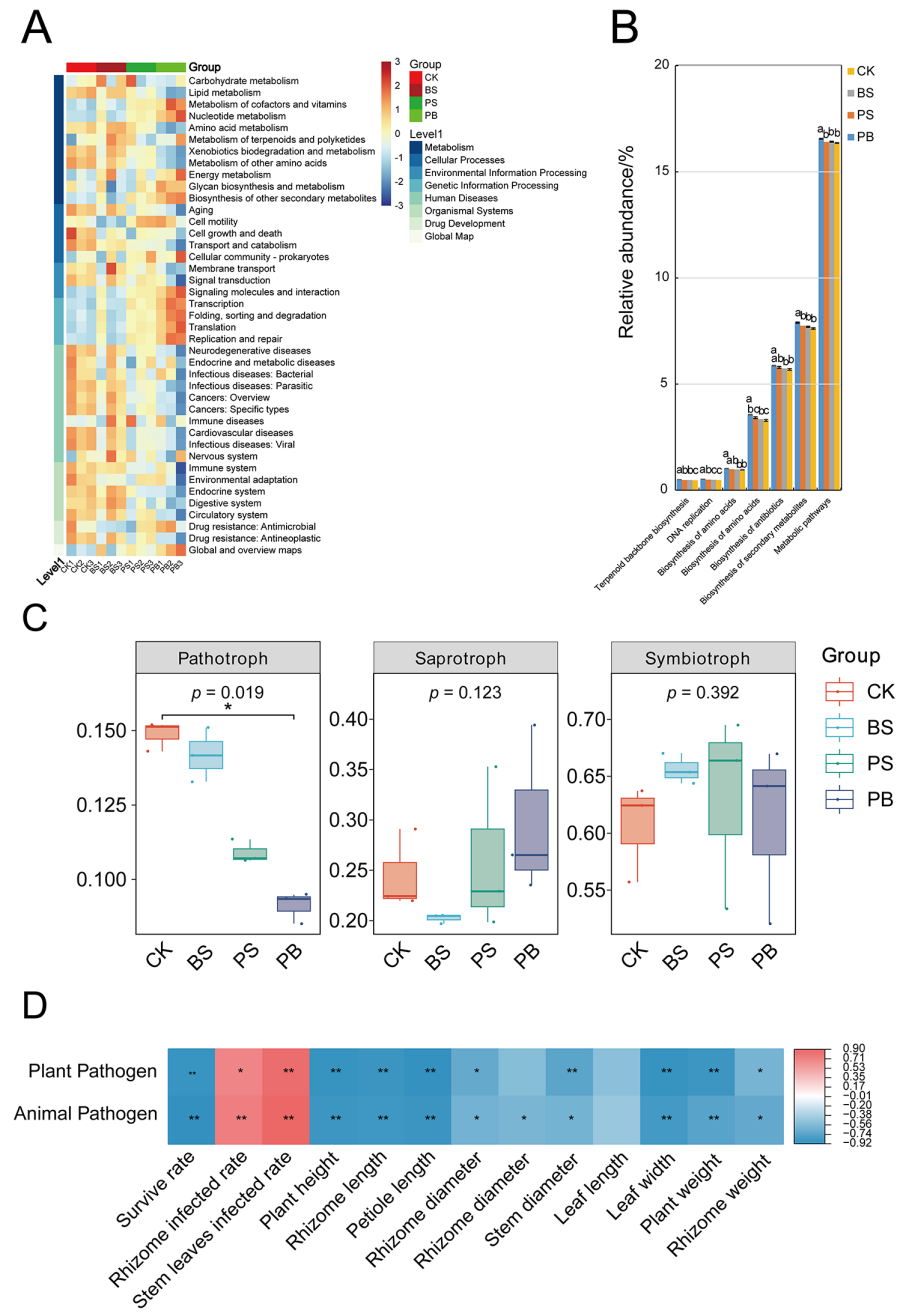
Predicted bacterial and fungi functions in ginseng rhizosphere soil under different treatments. (**A**) level1, 2 relative abundance clustering heatmap, (**B**) level3 significant difference histogram. Fungi community function predicted by FUNGuild, (**C**) level1 significant different box plots (one-way ANOVA, Tukey multiple comparisons test, *p* > 0.05), (**D**) Heatmap of correlation between fungi community function and disease index and agronomic traits. Different letters above the bars indicate significant differences between treatments at *p* < 0.05(*p < 0.05;** *p* < 0.01)(*n* = 3)

To gain insights into the direct effects of different treatment conditions on soil microbial function, 16 S rRNA gene sequences were predicted using PICRUSt. Eight classes of biometabolic pathways were analysed and systematically classified into two or three layers within each class of metabolic pathways. The results, show a comparison of KEGG pathway abundance between the three treatment groups, indicating that the PB treatment group exhibited higher metabolism, environmental information processing, global, and overview map function and lower human diseases (Fig. 8A, Table S14). Analysed at level 3, abundance of seven related functions including metabolic pathways (16.56%), biosynthesis of secondary metabolites (7.90%), biosynthesis of antibiotics (5.87%), biosynthesis of amino acids (3.56%), carbon fixation pathways in prokaryotes (1.02%), DNA replication (0.52%), and terpenoid backbone biosynthesis (0.48%) in ginseng rhizosphere soil, these seven functions were significantly higher in the group PB than in the control group (*p* < 0.05) (Fig. 8B). The Pp-7250 co-bacterial agent promoted ginseng growth, ginsenoside accumulation, pesticides residue degradation, and reduced ginseng disease rate by regulating the function of ginseng rhizosphere soil bacterial communities.

#### Fungi community function in ginseng rhizosphere soil treated with Pp-7250 co-bacterial agent

FUNGuild was used to predict the nutritional and functional groups of the fungal communities with different treaments. The four treatments had different trophic modes, including pathotroph, saprotroph and symbiotroph (Fig. 8C). The relative abundance of pathotroph was significantly reduced in the group PB compared to the group CK (*p* < 0.05), whereas the differences in the relative abundance of saprotroph and symbiotroph were not significant among the groups (*p* > 0.05). Further analysing the 27 categories of the three nutritional patterns, plant pathogens and animal pathogens were significantly lower in the group PB than in the other groups (*p* < 0.05). Plant pathogens and animal pathogens were significantly positively correlated with disease (*p* < 0.05), and were significantly negatively correlated with growth potential (plant height, fresh weight, etc.) (*p* < 0.05). (Fig. 8D, Table S15). The Pp-7250 co-bacterial agent promoted ginseng growth, ginsenoside accumulation, pesticides residue degradation, and reduced ginseng disease rate by regulating the function of ginseng rhizosphere soil fungal communities.

## Discussion

### Pp-7250 co-bacterial agent promotes ginseng yield enhancement, disease control, ginsenoside accumulation, and pesticides residue degradation

Applying microbial agents increases ginseng yield and has a significant impact on the ginseng industry. [16]. The present study suggested that PB co-bacterial agent treatment altered the microbial taxa in ginseng rhizosphere soil, such as *Bacillus* (increased by 602.59%) and *Paenibacillus* (increased by 1537.07%), which may explain why applying PB co-bacterial agents significantly boosted ginseng production performance. According to one report, a *Pseudomonas* and *Bacillus* co-bacterial agent promotes the growth of soybean and increases its yield [40]. The application of a mixture of *Bacillus* strains YH-18 and YH-20 can effectively improve the nutrient utilization of continuous cropping soils, distress the pathogenic bacteria of plants, and benefit the plant growth [41]. Mixed bioinoculant MI from *Bacillus*, *Pseudomonas*, *Streptomyces*, and *Trichoderma* can potentially enhance wheat growth and seed yield [42]. The present study demonstrated that the application of PB co-bacterial agents could provide an environmentally friendly and novel strategy to improve ginseng yield.

Bioinoculants are widely used to make plants more effective against diseases [43]. The present study demonstrated that the application of PB co-bacterial agents could be more effective in reducing disease incidence and improving ginseng survival rate. Many studies have demonstrated that combinations of microbial agents have superior biocontrol effects on plants. *B. subtilis* and *Pseudomonas fluorescens* showed synergistic biocontrol effects against early blight disease in tomatoes [44]. Using three strains, *B. subtilis* IN937b, *B. pumilus* SE34, and *B. amyloliquefaciens* IN937, simultaneously acted against the cucumber mosaic virus in tomato plants under field conditions as seed treatments. Therefore, we hypothesized that PB could be used as a novel and effective biocontrol agent against ginseng diseases.

Microorganisms promote metabolite accumulation in medicinal plants [45]. Ginseng endophytes are associated with ginsenoside accumulation, which is of great significance for improving ginseng quality [35]. The present study showed that microbial agents significantly increased the accumulation of ginsenoside content, with the most significant increase observed with the PB co-bacterial agent. Different microbial agents promoted an increase in different ginsenoside monomers at different levels, and the PB group had the highest accumulation of ginsenoside Rf compared to other monomers. It was previously shown that the microbial inoculants MI (B. *licheniformis*, *B. amylolyticus*, *B. subtilis*, *actinomycetes*, and yeast) could promote the accumulation of ginsenosides [16]. However, there are no reports that *P. polymyxa* and *B. cereus* co-bacterial agent application can promote the accumulation of secondary metabolites in medicinal plants. The results of this study implied that Pp-7250 co-bacterial agent application could promote the synthesis of ginsenosides. Therefore, we hypothesized that Pp-7250 co-bacterial agent could be used as an inducer of ginsenoside biosynthesis. However, whether Pp-7250 co-bacterial agent directly leads to the increase of ginsenoside content in ginseng remains to be further investigated in subsequent experiments.

Microbial degradation of ginseng pesticide residues has been widely studied. In this study, Pp-7250 co-bacterial agent application showed high efficiency in degrading PCNB and heptachlor pesticide residue in ginseng rhizosphere soil. The degradation rate of *Pseudomonas fluorescens* KT3 and *Bacillus subtilis* 2M6E combined bacteriological agent has been reported to be more than 95% for acetochlor and close to 100% for 2-methyl-6-ethylaniline. Although *Bacillus subtilis* 2M6E could not degrade acetochlor, it improved the degradation efficiency of *Pseudomonas fluorescens* KT3 [46]. *Bacillus subtilis* MK101, *Pseudomonas kermanshahensis* MK113 and *Rhodococcus fascians* MK144 were are effective azoxystrobin degraders [47]. *Ochrobactrum* sp. DGG-1-3, *Ochrobactrum* sp. Ge-14, *Ochrobactrum* sp. B18 and *Pseudomonas citronellolis* ADA-23B were more effective than the pure strains at degrading 2,4-D, carbofuran, and diazinon in a mixed combination [48]. Therefore, the combination of microbial agents improved the degradation rate of pesticide residues. We hypothezised that Pp-7250 co-bacterial agent could be used as a pesticide residue degrader for ginseng and its soil.

### Effect of Pp-7250 co-bacterial agent treatment on ginseng rhizosphere soil microbial communities

Microbial fungicides are important role in regulating the microbial diversity and community composition in plant rhizosphere soils [49]. In this study, we found that Pp-7250 co-bacterial agent application significantly reduced the α-diversity of fungi communities.The reason for the significant effects of PB co-bacterial agents application on ginseng disease index, agronomic traits, efficacy components, and pesticide residue degradation was related to the reduction of α-diversity of fungi communities. Microbial agents can provide prerequisites for maintaining plant health, growth, secondary metabolism and pesticide residues by improving the structure and function of soil microbial community composition [50].

The composition and abundance of microbial communities play an important role in soil microecological balance [51]. In terms of bacterial community composition, our study found that *Ascomycetes* and *Acidobacteria* were the dominant phyla in each group of ginseng soils, which is consistent with the results of the previous studies. *Ascomycetes* and *Acidobacteria* may play rolea in the maintaining of soil microbial homeostasis [52]. The relative abundance of *Gemmatimonadota* was significantly higher in the group PB than in the group CK (*p* < 0.05) (Table S10), and the benefits of *Gemmatimonadota* for ginseng are related to its ability to degrade organic substances to provide plant with nutrients such as N and P, thereby promoting the structural stability of soils, increasing the aeration and water retention of soils, improving soil quality, synthesizing beneficial substances for plant growth and health such as growth factors and antibiotics, and decomposing harmful substances such as pesticides in the soil [53, 54]. The relative abundance of *Basidiomycota* was significantly higher in the group PB than that in the group CK (*p* < 0.05) (Table S11). *Basidiomycota* are often symbiotic with plants to form mycorrhiza, which is beneficial for crop cultivation. The PB group was beneficial to the cultivation of ginseng through by promoting of higher numbers of *Basidiomycota* [55]. *Ascomycota* had the lowest abundance in PB group (Table S11). *Ascomycota* often causes sclerotinia, root rot, and black spots in plant [56]. The PB group improved ginseng health by suppressing the number of *Ascomycota* pathogens.

The relative abundance of potentially beneficial microorganisms genera in ginseng soil was significantly increased by PB agent application (*p* < 0.05), such as *Rhodanobacter*, *Pseudolabrys*, *Gemmatimonas*, *Bacillus*, *Paenibacillus*, and *Russula* (Fig. 5). *Rhodanobacter* was significantly positively correlated with root diameter and leaf length, proving that *Rhodanobacter* promoted ginseng growth. *Rhodanobacter* is a good biological control over *Cylindrocladium spathiphylli* [57]. *Rhodanobacter* combined with *Achromobacter*, *Castelaniella*, and *Hypomicrobium* for completely degraded phenanthrene and pyren at 10 and 15d, respectively [58]. *Rhodanobacter* degraded the diisobutyl phthalate (DiBP) produced by ginseng replantation problem (GRP) [49]. It is hypothesized that increasing *Rhodanobacter* is responsible for the improved biocontrol and pesticides residue degradation effects of Pp-7250 co-bacterial agent application. *Pseudolabrys* was positively correlated with the content of ginsenosides Rb1 and Rc [59]. It is hypothesized that the increase in *Pseudolabrys* is one of the reasons for the increased accumulation of ginsenosides following applying the PB co-bacterial agent. Members of the *Gemmatimonas* contribute to soil organic carbon fixation [60], and it has a variety of roles such as fighting plant pathogens, improving soil nutrients (converting insoluble P to soluble P), fighting oxidative stress, and promoting plant growth [61]. *Gemmatimonas* is more abundant in the rhizosphere soil of healthy plants than in diseased soils [62], and *Gemmatimonas* can induce plant resilience or produce antifungal antibiotics [63, 64], suggesting its plant disease suppression properties. *Gemmatimonas* may be involved in glyphosate biodegradation [65]. In this study, *Gemmatimonas* showed increased abundance in PB treatments, and *Gemmatimonas* was significantly positively correlated with ginseng root length, stem diameter, biocontrol effect, and ginsenoside accumulation and significantly negatively correlated with PCNB pesticide residue. Therefore, *Gemmatimonas* has an important role in ginseng cultivation. *Bacillus* spp. can produce growth hormones, antibiotics, antimicrobial proteins, and other functional substances that promote plant growth, restrain pathogenic bacteria, facilitate pesticide degradation, and increase crop yield [66–68]. Intercropping potato and tomato alters the composition of the tomato rhizosphere microbiome by promoting the colonization of *Bacillus* species, thereby inhibiting the growth of fungi *Verticillium* and enhancing the resistance of tomato plants. [69]. *Bacillus* is directly associated with the transfer of resources at the expense of biomass to enhance plant growth and induce plant defenses, but its abundance is relatively lower than that of most of the major endophytic bacteria and is very low in all plants. The present study demonstrated that *Bacillus* were significantly and positively associated with ginseng yield, content of nine ginsenosides, biodefense, and pesticide degradation. It is hypothesized that Pp-7250 co-bacterial agent application to increases the abundance of *Bacillus*, which is responsible for the growth promotion, biological control, and pesticides residue degradation effects in ginseng. *Paenibacillus* not only has good applications in biological control [25, 58], but also promotes ginseng growth and the conversion of ginsenosides and five pesticides: fluazinam, BHC, PCNB, chlorpyrifos, and DDT [21, 70]. In the present study, the effects of *Paenibacillus* on ginseng yield, relative defense efficacy, and the content of the nine ginsenosides, and pesticides residue degradation were significantly and positively correlated. It was hypothesized that applying the PB co-bacterial agent increased *Paenibacillus* abundance, which was responsible for promoting ginseng growth, biocontrol, ginsenosides accumulation, and pesticide residue degradation. *Russula* produces bioactive compounds with antimicrobial and antioxidant properties [71]. *Russula vinosa* promotes crop growth and exerts toxic effects on plant pathogens [72]. Similar results were obtained in this study.

In this study, *Fusarium*, *Tetracladium*, *Alternaria*, and *Ilyonectria* were significantly reduced in the ginseng rhizosphere soil treated with PB co-bacterial agents (*p* < 0.05). *Fusarium* and *Ilyonectria* are the main causes of root rot in ginseng, which leads to decreased yield and poor growth [73, 74]. *Ilyonectria* produces specific metabolites, such as resorcylic acid lactones, which affect the plant immune system [75]. *Ilyonectria* is a highly pathogenic fungus for ginseng, often causing erythroderma, and root rot [76]. *Ilyonectria* and *Tetracladium* indicators of soil pathogenicity [77], increased significantly with the years of cultivation [78], exacerbating the development of soil-borne diseases and increasing mortality [79]. *Alternaria* is a pathogenic fungus associated with ginseng root rust [80]. Ginseng black spot disease is caused by *Alternaria* ginsengensis [81]. In this study, these pathogens were negatively correlated with biological defense efficacy and agronomic traits (*p* < 0.05). It was hypothesized that the Pp-7250 co-bacterial agent increased ginseng survival and root yield, while reducing ginseng disease incidence by suppressing the pathogen abundance of *Fusarium*, *Tetracladium*, *Alternaria*, and *Ilyonectria*.

### Effect of Pp-7250 co-bacterial agent treatment on the functioning of soil microbial communities in ginseng rhizosphere

Currently, PICRUSt and FUNGuild analyses are been applied to the study of ginseng rhizosphere soil bacterial and fungal functions, linking changes in microbial biological functions to microbial community functions [82]. The results of functional analyses of bacterial communities in this study indicated that the increased function of the group PB metabolic pathways provides conditions for ginseng growth and pesticides residue degradation [83]. Biosynthesis of amino acids pathways provides a basis for increasing plant height and protein content [84], and biosynthesis of secondary metabolites and terpenoid backbone biosynthesis, validating the the potential for biosynthesis of ginsenosides and biocontrol by *Bacillus* and *Paenibacillus* [85] and showing that carbon fixation pathways in prokaryotes and the DNA replication pathway are necessary pathways for ginseng under the action of growth [86]. Combined with the analysis of the differential bacterial composition, the authors concluded that an increase in potentially beneficial bacteria, such as *Rhodanobacter*, *Pseudolabrys*, *Gemmatimonas*, *Bacillus*, *Paenibacillus*, *Cortinarius*, *Russula*, *Paecilomyces*, and *Trechispora* was responsible for ginseng resistance to disease, improved yield and quality, and pesticide degradation. Previous studies have demonstrated that the genes that undergo significant changes after microbial inoculation are mainly metabolism-related functional genes, suggesting that metabolic functions play an extremely important role in soil microbial ecology [87]. The dominant trophic pattern of fungi changed with the relative abundance of fungi. The relative abundance of soil pathological trophic phenotypes in the ginseng rhizosphere treated with the PB co-bacterial agent was significantly reduced, and the group PB had significantly lower plant pathogens and animal pathogens than all other groups (*p* < 0.05). The abundance of plant pathogens and animal pathogens was significantly positively correlated with ginseng diseases and significantly negatively correlated with ginseng yield (plant height and weight). Combined with the analysis of differential composition of fungi, we concluded that applying the PB co-bacterial agent had a direct effect on the abundance of the fungal community, and that the reduction of *Fusarium*, *Tetracladium*, *Alternaria*, and *Ilyonectria* was the reason for the suppression of ginseng diseases by the Pp-7250 co-bacterial agent.

## Conclusions

The present study elucidated, for the first time, the effect of PB co-bacterial agents on the yield and quality of ginseng. PB treatment increased ginseng yield, ginsenoside accumulation, disease prevention, and pesticide degradation. The PB treatment promoted an increase in the beneficial microorganisms, including *Rhodanobacter*, *Pseudolabrys*, *Gemmatimonas*, *Bacillus*, *Paenibacillus*, *Cortinarius*, *Russula*, *Paecilomyces*, and *Trechispora* in ginseng rhizosphere soils and a decrease in pathogenic microorganisms, including *Ellin6067*, *Acidibacter*, *Fusarium*, *Tetracladium*, *Alternaria*, and *Ilyonectria*. The Pp-7250 co-bacterial agent improved the function of microbial metabolic pathways, biosynthesis of secondary metabolites, biosynthesis of antibiotics, biosynthesis of amino acids, carbon fixation pathways in prokaryotes, DNA replication, and terpenoid backbone biosynthesis, and decreased the function of microbial plant pathogens and animal pathogens. This study provides a theoretical basis for improving the synergistic effect of microbial phytopathogens in combination, which is of great practical value for improving the yield and quality of planted ginseng.

### Electronic supplementary material

Below is the link to the electronic supplementary material.

**Supplementary Material 1: Table S1**. Statistical results of bacterial sequencing data processing

**Supplementary Material 2: Table S2**. Statistical results of fungi sequencing data processing

**Supplementary Material 3: Table S3**. Analysis of ginseng disease incidence by application of Pp-7250 co-bacterial agent

**Supplementary Material 4: Table S4**. Analysis of ginseng agronomic traits by application of Pp-7250 co-bacterial agent

**Supplementary Material 5: Table S5**. Analysis of 9 ginsenosides accumulative by application of Pp-7250 co-bacterial agent (%)

**Supplementary Material 6: Table S6**. Analysis of ginseng pesticide residue content by application of Pp-7250 co-bacterial agent (mg/kg)

**Supplementary Material 7: Table S7**. Analysis of ginseng pesticide residue degradation rate by application of Pp-7250 co-bacterial agent (%)

**Supplementary Material 8: Table S8**. Classification of ginseng rhizosphere soil bacterial OTUs at the phylum and species levels

**Supplementary Material 9: Table S9**. Classification of ginseng rhizosphere soil fungal OTUs at the phylum and species levels

**Supplementary Material 10: Table S10**. Relative abundance at phylum level of the top 15 bacteria (%)

**Supplementary Material 11: Table S11**. Relative abundance at phylum level of the top 15 fungi (%)

**Supplementary Material 12: Table S12**. Relative abundance at genus level of the top 20 bacteria (%)

**Supplementary Material 13: Table S13**. Relative abundance at genus level of the top 20 fungi (%)

**Supplementary Material 14: Table S14**. Bacterial community function level1, 2 Relative Abundance (%)

**Supplementary Material 15: Table S15**. Correlation analysis of fungi functions with ginseng diseases and growth indicators

## Data Availability

P. polymyxa Pp-7250 (Pp-7250) is preserving in the General Microbiological Center of the China Microbial Culture Collection Management Committee, Preservation number CGMCC: No.7250 (http://www.cgmcc.net). The 16S RNA gene sequences of B. cereus are available through the National Center of Biotechnology Information under accession number: PP556973.(https://www.ncbi.nlm.nih.gov/nucleotide/PP556973). The raw fastq data of 16S and ITS rRNA can be available at NCBI Sequence Read Archive (SRA) website database using the given NCBI accession number PRJNA1093352 (http://www.ncbi.nlm.nih.gov/bioproject/1093352), PRJNA1093355 (http://www.ncbi.nlm.nih.gov/bioproject/1093355). All data analyzed during this study are included in this published article and its additional files.
